# Deconvolution of the Gene Expression Profiles of Valuable Banked Blood Specimens for Studying the Prognostic Values of Altered Peripheral Immune Cell Proportions in Cancer Patients

**DOI:** 10.1371/journal.pone.0100934

**Published:** 2014-06-24

**Authors:** Lishuang Qi, Bailiang Li, Yu Dong, Hui Xu, Libin Chen, Hongwei Wang, Pengfei Li, Wenyuan Zhao, Yunyan Gu, Chenguang Wang, Zheng Guo

**Affiliations:** 1 College of Bioinformatics Science and Technology, Harbin Medical University, Harbin, China; 2 College of Pharmacy, Harbin Medical University, Harbin, China; 3 Department of Bioinformatics, School of Basic Medical Sciences, Fujian Medical University, Fuzhou, China; National University of Ireland Galway, Ireland

## Abstract

**Background:**

The altered composition of immune cells in peripheral blood has been reported to be associated with cancer patient survival. However, analysis of the composition of peripheral immune cells are often limited in retrospective survival studies employing banked blood specimens with long-term follow-up because the application of flow cytometry to such specimens is problematic. The aim of this study was to demonstrate the feasibility of deconvolving blood-based gene expression profiles (GEPs) to estimate the proportions of immune cells and determine their prognostic values for cancer patients.

**Methods and Results:**

Here, using GEPs from peripheral blood mononuclear cells (PBMC) of 108 non-small cell lung cancer (NSCLC) patients, we deconvolved the immune cell proportions and analyzed their association with patient survival. Univariate Kaplan-Meier analysis showed that a low proportion of T cells was significantly associated with poor patient survival, as was the proportion of T helper cells; however, only the proportion of T cells was independently prognostic for patients by a multivariate Cox regression analysis (hazard ratio = 2.23; 95% CI, 1.01–4.92; *p* = .048). Considering that altered peripheral blood compositions can reflect altered immune responses within the tumor microenvironment, based on a tissue-based GEPs of NSCLC patients, we demonstrated a significant association between poor patient survival and the low level of antigen presentation, which play a critical role in T cell proliferation.

**Conclusions:**

These results demonstrate that it is feasible to deconvolve GEPs from banked blood specimens for retrospective survival analysis of alterations of immune cell composition, and suggest the proportion of T cells in PBMC which might reflect the antigen presentation level within the tumor microenvironment can be a prognostic marker for NSCLC patients.

## Introduction

Immune responses to tumor cells in the tumor microenvironment play critical roles in the determination of tumor progression [1], [2]. A better understanding of these immune responses could reveal immune-related markers that could be used to stratify cancer patients with different risks of recurrence or death [3], [4]. For example, high densities of tumor-infiltrating lymphocytes and dendritic cells are associated with prolonged survival in a variety of malignancies, including non-small cell lung cancer (NSCLC) [5], [6], [7], colorectal cancer [8], [9], and ovarian cancer [10], [11]; attributed to their fundamental roles in anti-tumor immunity. It is known that immune cells respond dynamically to variations in the tumor microenvironment [12], [13], thus continuously monitoring these varying immune responses might facilitate the prevention of cancer recurrence or deterioration by enabling treatment protocols to be modulated in a timely manner. However, the immune-related markers in the tumor microenvironment can only be assessed once, at the time of surgical resection for operable cases. To overcome the defect of tumor tissue analysis, the prognostic value of alterations of the immune cell composition in the peripheral blood could be investigated; changes in the immune cell composition reflect the complicated immune status within the tumor microenvironment [14], [15], [16] and would continue to be measureable after surgical resection. In fact, many studies have found that altered compositions of peripheral immune cells, such as lymphocyte proportion, neutrophil proportion, and neutrophil-to-lymphocyte ratios in the peripheral blood, are potential markers for survival in cancer patients, including those with NSCLC [17], [18], colorectal cancer [19] and early gastric cancer [20].

To identify potential prognostic markers, retrospective studies are widely used [21], [22], [23] because the long-term follow-up of cancer patients is rather expensive and time-consuming [24]. Potential prognostic markers are usually validated using prospective studies for clinical application [25]. Tissue or blood specimens are typically accumulated on a long-term basis until there are clear objectives for study. For example, when recent investigations suggest that changes in gene expression or epigenetic may carry information that is predictive of patient outcomes, previously tissue or blood banked specimens with long-term follow-up can be used for microarray analysis to explore prognostic gene or methylation panels [16], [26], [27]. However, it is problematic to measure the proportions of immune cells in such long-term banked blood specimens by flow cytometry which is routinely applied to fresh blood specimens [28], because the specific surface proteins of immune cells may be unstable during long-term cryopreservation [29]. Therefore, other methods for evaluating the proportions of various immune cells in such valuable accumulated blood specimens must be explored for retrospective survival analysis.

Several groups have recently proposed deconvolution methods to estimate the proportions of immune cells in peripheral blood based on gene expression profiles and marker genes that are specifically expressed in immune cells [30], [31]. The proportions estimated by the deconvolution methods are highly correlated with actual proportions in heterozygous blood specimens. For example, the deconvolution method proposed by Gaujoux et al. was previously validated on a microarray dataset of heterogeneous specimens with known proportions of the four cell types (Jurkat, IM-9, Raji, THP-1) [31]. It was found that the mean absolute difference and the pearson correlation coefficient between the actual and estimated proportions were 0.05 and 0.91, respectively. Advantageously, gene expression in mRNA from peripheral whole blood or peripheral blood mononuclear cells (PBMC) is relatively stable under cryopreservation conditions because low temperatures inhibit RNA degradation [32], making deconvolution methods applicable to banked blood specimens for retrospective survival analysis. Deconvolution methods have been applied to the study of some immune diseases, such as systemic lupus erythematosus [30], but not to any cancer research.

In this study, we used the PBMC gene expression profiles of 108 NSCLC patients to demonstrate the feasibility of a deconvolution method for studying the prognostic value of altered peripheral blood composition in cancer patients with banked blood specimens. First, we evaluated the proportions of various immune cells using a deconvolution method and found that the proportion of T cells was an independent prognostic marker for NSCLC patients by a multivariate Cox regression analysis, after adjusting for the other potential prognostic factors that were significant in the univariate analysis. We then obtained a tentative evidence supporting the assumption that the low level of antigen presentation in the tumor microenvironment might be a major cause of the decreased T cell proportion in the peripheral blood by demonstrating a consistent association between the low level of antigen presentation and poor patient outcomes.

## Materials and Methods

### Microarray data

The dataset of gene expression profiles taken pre-surgery from the PBMC of 108 NSCLC patients with survival information was downloaded from the NCBI Gene Expression Omnibus (GEO; http://www.ncbi.nlm.nih.gov/geo/; series accession number GSE13255) [33]. The dataset was generated by the Genome Illumina human-6 v2.0 expression beadchip; the arrays in the dataset were quantile normalized, and the background was subtracted from the expression values, as previously described [34]. The probe sets were annotated using the GPL6102 data file. Probe sets that did not match any gene ID and those that matched multiple gene IDs were deleted. For each sample, the expression intensities of the probe sets that matched the same gene ID were averaged as the expression intensity of that gene ID. The clinical characteristics of patients were summarized in Table 1. As it has been suggested that NSCLC patients are largely insensitive to adjuvant chemotherapy [35], [36], all of the patients were considered together for the following analysis.

**Table 1:** Clinical characteristics of the NSCLC patients in the PBMC dataset.

Variable	Patients [N]	[%]
**All**	108	100
**Histology**		
AD	67	62
SCC	34	31
NSCLC	7	7
**Gender**		
Male	53	49
Female	55	51
**Stage**		
I	66	61
II & III	42	39
**Age (years)**		
<68	49	45
≥68	59	55
**Race**		
C	99	92
AA	9	8
**Smoke**		
Never	6	5
Formerly	87	81
Currently	15	14
**Chemotherapy**		
No	52	48
Yes	34	32
Not sure	22	20
**COPD**		
Yes	54	50
No	50	46
Not sure	4	4

**Notes:** “AD” and “SCC” represent adenocarcinoma and squamous cell carcinoma, respectively; “C” and “AA” represent Caucasian and African American; “COPD” represents chronic obstructive pulmonary disease.

A tissue-based gene expression dataset of NSCLC (GSE11969) with comprehensive clinical characteristics was selected to study the association between the antigen presentation level in tumor tissues and patient survival. The methods used to generate and normalize the dataset were described previously [37]. Table S1 summarized the clinical characteristics of the patients in the tissue-based dataset.

### Evaluating the proportions of immune cells in PBMC

Based on the PBMC gene expression profiles of the 108 NSCLC patients and the marker genes specifically expressed on B lymphocytes, T lymphocytes, natural killer (NK) lymphocytes, dendritic cells (DCs) and monocytes (excluding monocyte-derived DCs) documented in the Immune Response in Silico (IRIS) database [38], we evaluated the proportions of the five immune cells, which together make up 100% of PBMC, using a modified semi-supervised nonnegative matrix factorization method for gene expression deconvolution [31].

The algorithm assumes that the expression intensity of a gene in a sample can be modeled as a linear combination of the expression intensities of that gene in all cell types comprising that sample. Briefly, the expression intensity of the *i*th gene in the *j*th sample is the sum of the *i*th gene expression intensities in all *r* cell types present in the sample:
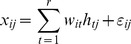
(1)where *w_it_* is the expression intensity of the *i*th gene in the *t*th cell type and *h_tj_* is the proportion of the *t*th cell type in the *j*th sample; *ε_ij_* is a random error.

Given a nonnegative global gene expression matrix *X* of *n* genes in *p* samples, the ssKL algorithm aims to find an approximate matrix decomposition equation:
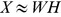
(2)where *W* is the *n*×*r* matrix representing the gene expression profiles of all *r* cell types and *H* is the *r*×*p* matrix representing the proportion profiles of all the *r* cell types in the heterogeneous samples.

Similarly, we estimated the proportions of various immune cells in PBMC based on the marker genes of immune cells characterized by HaemAtlas [39], which classifies T cells into T helper lymphocytes (Th) and cytotoxic T lymphocytes (CTL) and also includes B cells, NK cells, and monocytes (including DCs and other monocytes) using the deconvolution method.

All calculations were performed using the CellMix package in R 2.15.3 software [40].

### Survival analysis

Overall survival (OS) was defined as the time from the date of initial surgical resection to the date of death or last contact (censored). For the PBMC dataset, we classified the patients into two groups (Low vs. High) based on the median proportion of each immune cell among all samples. OS was estimated by a univariate analysis using the Kaplan-Meier method, and the OS difference between groups was determined using the log-rank test [41]. The associations between clinical factors and OS were also analyzed using the univariate Kaplan-Meier analysis; the examined factors included histological subtype (adenocarcinoma vs. squamous cell carcinoma), gender (male vs. female), tumor stage (II–III vs. I), age (≥68 years vs. <68), Race (Caucasian vs. African American), smoking status (formerly vs. currently), adjuvant chemotherapy (yes vs. no), and COPD status (present vs. absent). For the prognostic factors that were found to be significant in the univariate analysis, multivariate Cox regression analysis [42] was performed to determine the independent prognostic factors. Significance was defined as a *p* value<.05.

Similarly, for the tissue-based dataset, we used the univariate Kaplan-Meier analysis and multivariate Cox regression analysis to evaluate the association between OS and the antigen presentation level (Low vs. High) as well as clinical factors, including tumor stage (II–III vs. I), age (≥62 years vs. <62 years), histological subtype (adenocarcinoma vs. squamous cell carcinoma vs. large cell carcinoma) and gender (male vs. female). The antigen presentation level in the tumor microenvironment was characterized by the expression intensities of the major histocompatibility complex (*MHC*) genes through which DCs present the tumor antigen to T cell receptors [43]. We stratified the patients into two groups (Low vs. High) based on the expression intensities of the *MHC* genes using the K-means clustering algorithm with Euclidean distance between two samples, which was calculated as follows:
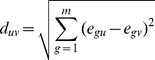
(3)where *m* is the number of *MHC* genes; *e_gu_* and *e_gv_* are the expression intensities of the *g*th gene in the *u*th and *v*th samples, respectively.

## Results

### Association between the proportions of immune cells in PBMC and OS

**Figure 1:**
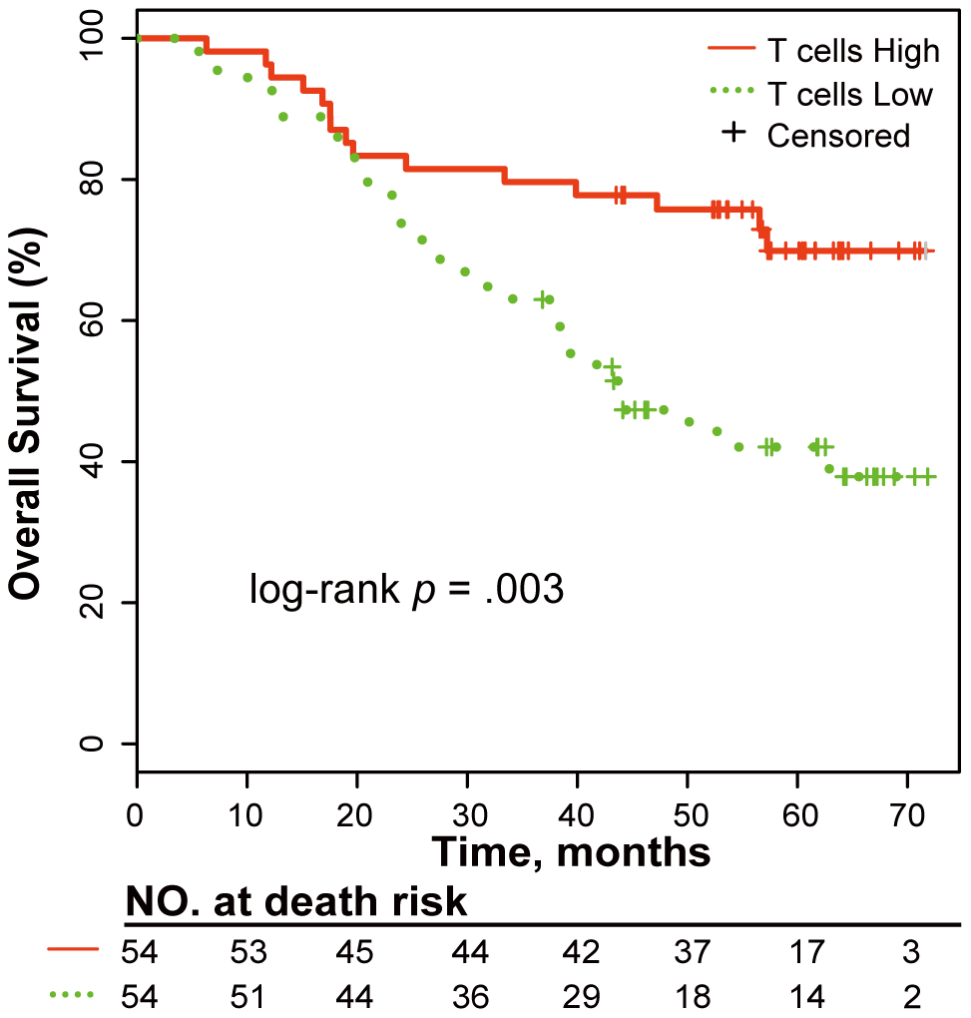
Kaplan-Meier curves for patients stratified by the proportion of T cells. The patients were stratified into two groups based on the median of the T cell proportion among the NSCLC patients: Low (less than the median) and High (greater than or equal to the median). The median overall survival (OS) was assessed using the Kaplan-Meier analysis. *p* was calculated using the log-rank test.

First, based on the marker genes specifically expressed on B cells, T cells, NK cells, DCs, and monocytes documented in the IRIS database, we adopted the modified semi-supervised nonnegative matrix factorization method to estimate the proportions of the immune cell types in each of the 108 NSCLC patients for which PBMC expression profiles were available (see Materials and Methods). For each type of immune cell, we stratified the patients into two groups, Low (below the median) and High (equal to or greater than the median), according to the median of the cell proportion among the patients and then tested the difference in OS between the groups using the univariate Kaplan-Meier analysis. The OS of the patients in the T cells Low group was significantly worse than that of the patients in the T cells High group (log-rank *p* = .003; Fig. 1). The proportions of B cells, NK cells, DCs, and monocytes were not observed to be associated with the OS of NSCLC patients (log-rank *p*>.05; Table 2).

**Table 2:** Survival analysis of patients with NSCLC based on the PBMC dataset.

	Univariate	Multivariate		
Variable	log-rank *p* a	HR	95%	*p* b
**B group (L vs. H)** *	.127	–	–	–
**T group (L vs. H)** *	.003	2.23	1.01–4.92	0.048
**NK group (L vs. H)** *	.251	–	–	–
**DC group (L vs. H)** *	.088	–	–	–
**Monocyte group (L vs. H)** *	.185	–	–	–
**Th group (L vs. H)** **	.009	1.04	0.48–2.28	0.92
**CTL group (L vs. H)** **	.061	–	–	–
**Histology (LCC vs. AD)**	.371	–	–	–
**Gender (male vs. female)**	.738	–	–	–
**Stage (II–III vs. I)**	.002	1.95	1.06–3.57	0.031
**Age (≥68 vs. <68)**	.012	2.01	1.05–3.86	0.036
**Race (C vs. AA)**	.309	–	–	–
**Smoking (formerly vs. currently)**	.81	–	–	–
**Adjuvant chemotherapy (yes vs. no)**	.186	–	–	–
**COPD (present vs. absent)**	.2	–	–	–

Note:

a The log-rank *p* value was derived from the Kaplan-Meier method using the log-rank test;

b the *p* value was derived from the Cox regression model; HR, hazard ratio; CI, confidence interval;

* The cell proportions estimated using marker genes in the IRIS, L, group with a lower cell proportion (<median), H, group with a higher cell proportion (≥median).

** The cell proportions estimated using marker genes in the HaemAtlas.

**Figure 2:**
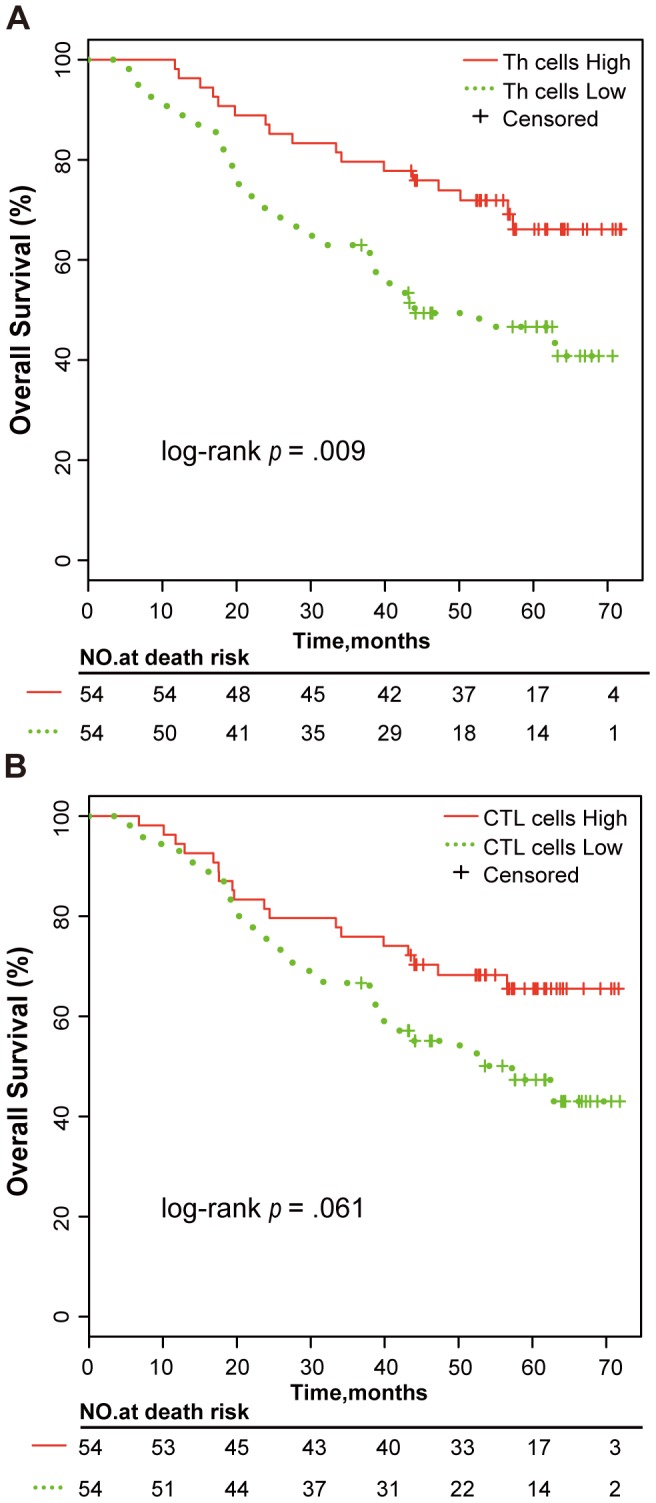
Kaplan-Meier curves for patients stratified by the proportion of T cell subsets. The patients were stratified into two groups according to the median of (A) Th cell proportion and (B) CTL cell proportion in PBMC among the NSCLC patients.

Then, based on the marker genes of the immune cells characterized by HaemAtlas, we estimated the proportions of Th and CTL cells, B cells, NK cells, and monocytes in PBMC and evaluated the associations of these proportions with OS using the univariate Kaplan-Meier analysis. The patients with a low proportion of Th cells (< median) had significant worse OS than those with a high proportion of Th cells (≥ median; log-rank *p* = .009; Fig. 2A), and patients with low proportion of CTL cells was marginally associated with worse OS (log-rank *p* = .061; Fig. 2B). The proportions of B cells, NK cells, and monocytes were not found to be significantly or marginally associated with the OS of NSCLC patients (log-rank *p*>.05; data not shown).

Taken together, these results suggest that the proportions of T cells and its subset Th cells are potential prognostic factors for NSCLC patients.

### The proportion of T cells in PBMC is an independent prognostic marker of OS

Multivariate Cox regression analysis was performed of the T cell proportion, Th cell proportion and the clinical factors that were found to be significant in the univariate analysis, including the age and tumor stage of patients. The proportion of T cells remained significantly associated with OS as an independent prognostic factor for NSCLC patients (T cells Low vs. T cells High groups: hazard ratio [HR] = 2.23, 95% CI, 1.07–4.21; *p* = .048). The age and tumor stage of patients were also independently prognostic for NSCLC patients in the multivariate model (Table 2).

### Association of tissue antigen presentation level with OS

One of the major sources leading to reduced proportion of T cells in PBMC might be the low antigen presentation level by DCs in the tumor microenvironment; DCs play a critical role in T cell proliferation in secondary lymphoid organ, from which the proliferated T cells migrate to peripheral blood [44]. Thus, we next determined whether the level of antigen presentation by DCs in tissues could predict patient outcome, consisting with T cell proportion in PBMC.

**Figure 3:**
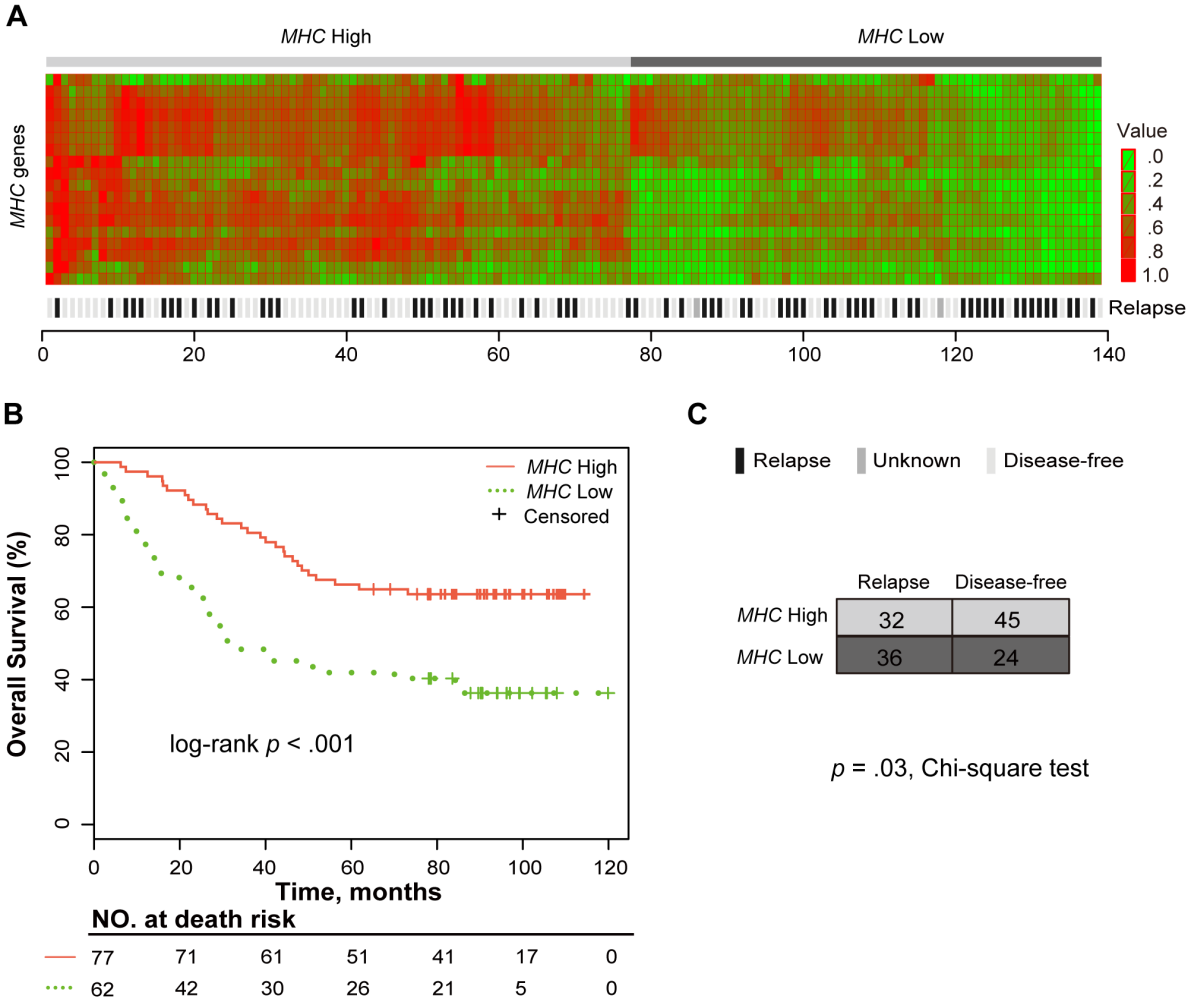
The *MHC* genes signature identifies two groups with different death and relapse risks. (A) Heat map of the *MHC* genes in the NSCLC patients. For the tissue expression dataset for NSCLC, the patients were classified into two *MHC*-related groups (*MHC* High and *MHC* Low) based on the expression intensities of 18 detected *MHC* genes listed in Table S2, using two-means clustering. The expression intensities of the *MHC* genes characterize the level of antigen presentation by DCs in the tumor microenvironment. (B) Kaplan-Meier curve for patients stratified into two *MHC*-related groups. The *p* value was calculated using the log-rank test. (C) The distribution of relapsing patients in the *MHC*-related groups. The chi-square test was used to compare the correlation between the level of *MHC* gene expression and relapse. Two patients were excluded because information about their relapse was unknown.

For the tissue-based expression profile dataset of 139 NSCLC patients, we applied the two-means clustering algorithm (see Materials and Methods) to stratify patients into two *MHC*-related groups based on the expression intensities of the *MHC* genes, which characterize the level of antigen presentation by DCs in the tumor microenvironment. The patients with a low expression pattern of *MHC* genes comprised the *MHC* Low group, while the remaining patients comprised the *MHC* High group, as shown in Fig. 3A. In the univariate Kaplan-Meier analysis, OS of the patients in the *MHC* Low group was significantly worse than that of the patients in the *MHC* High group (log-rank *p*<.001, Fig. 3B).

Next, we performed a multivariate Cox regression analysis of the level of antigen presentation, tumor stage and age, which were found to be significant in the univariate analysis. We found that the expression level of *MHC* genes was an independent prognostic factor for OS after adjusting for the tumor stage and age of patients (*MHC* Low vs. *MHC* High: HR = 2.45, 95% CI, 1.51–3.99; *p*<.001; Table 3). In addition, we found that the relapsing patients were significantly overrepresented in the *MHC* Low group (*p* = .03; chi-squared test; Fig. 3C).

**Table 3:** Survival analysis of NSCLC patients based on the tissue dataset.

	Univariate	Multivariate		
Variable	log-rank *p* a	HR	95% CI	*p* b
***MHC*** ** group (H vs. L)** *	3.0E-04	2.45	1.51–3.99	3.0E-04
**Stage (II–III vs. I)**	1.4E-08	2.15	1.63–2.84	5.4E-08
**Age (≥63 years vs. <62 years)**	.021	1.94	1.18–3.19	8.8E-03
**Histology (AD vs. SCC vs. LCC)**	.125	–	–	–
**Gender (male vs. female)**	.159	–	–	–

**Note:**

a The log-rank *p* value was derived from the Kaplan–Meier method using the log-rank test;

b the *p* value was derived from the Cox regression model; HR, hazard ratio; CI, confidence interval;

*L, the group with low *MHC* gene expression pattern; H, the group with high *MHC* gene expression pattern.

## Discussion

In this study, using the gene expression profiles of NSCLC patients, we demonstrated the feasibility of deconvolving proportions of peripheral immune cells to study their prognostic values for cancer patients using long-term banked blood specimens, which are important materials for retrospective survival analysis. The results suggested that the proportion of T cells in PBMC is a promising prognostic biomarker for NSCLC patients. Our analysis revealed that the low proportion of T cells was significantly associated with poor survival of NSCLC patients. In addition, the deconvolution method could be used to further explore the prognostic values of more refined immune cell subsets in cancer patients based on the same gene expression profiles. For instance, our analysis further demonstrated that the low proportion of one T cell subset, Th cells, was also significantly associated with survival of NSCLC patients. While the proportion of T cells was the only independent prognostic immune marker for overall survival after adjusting for other potential prognostic variables. As both of Th cells and CTL cells play major roles in anti-tumor immunity by specifically identifying tumor cells as “non-self” [45], the degree to which overall proportion of T cells decrease in peripheral blood may effectively reflect the degree of the deterioration of the immune response to tumor cells and thus may correlate closely with patient survival. Compared with the prognostic gene panels identified by gene expression profiles [16], [46], the T cell proportion in peripheral blood may be more acceptable as a prognostic marker for clinical application because of its easy detection and explicable biological principle.

The altered composition of peripheral immune cells might be the result of altered immune responses within the tumor microenvironment. Therefore, we assumed that one of the major sources leading to a reduced T cell proportion in PBMC could be a low level of antigen presentation by DCs in the tumor microenvironment of NSCLC patients, as DCs play a central role in T cell proliferation in secondary lymphoid organ, from which the proliferated T cells migrate to peripheral blood (and then to the tumor site) [44], [47]. Consistent with this assumption, our results revealed that the low expression level of *MHC* genes through which DCs present the tumor antigen to T cells [43], [48], in tumor tissues was also independently prognostic of poor survival in NSCLC patients. This result also supports a previous report that DCs dysfunction in tumor tissues, which is a critical mechanism for escaping the immune surveillance of tumor cells [48], is associated with poor survival in NSCLC patients [7], [49]. To further verify this assumption, we must simultaneously determine the gene expression profiles in tumor tissue, secondary lymphoid organ, and peripheral blood in the same cohort of NSCLC patients in future studies. This result also suggests that the outcomes of NSCLC patients with the low level of antigen presentation of DCs, could be improved by reinvigorating the immune status of DCs in the tumor microenvironment using immunotherapies such as DCs vaccines [50]. It is known that the tissue-based immune markers are limited and uncertain for clinical application as they could be influenced by the differences in tumor region, such as the center or the invasive margin of the tumor, of sampling from the cancer [51]. Detecting the prognostic markers (such as T cell proportion) in peripheral blood could avoid this problem. Additionally, because the peripheral prognostic markers can be easily detected in post-surgery blood specimens from cancer patients at regular intervals, they could also provide information to help physicians modulate treatment protocols for patients in a timely manner to improve outcomes. Therefore, the T cell proportion in peripheral blood, which might reflect the antigen presentation level in tumor tissues, may become a promising biomarker to predict the outcomes and monitor the progression for NSCLC patients.

Notably, the proportions of immune cells estimated by the deconvolution method are just positively correlated with the actual proportions in complex biological samples [31]. Thus, after using the deconvolution method to estimate immune cell proportions in banked blood specimens of patients and identify prognostic markers, we recommend that prospective studies should utilize flow cytometry to validate the results and determine the optimal cutoff values of the proportions for clinical application. Specifically, flow cytometry in large-scale prospective studies must be used to determine the clinical applicability of the T cell proportion in PBMC as a prognostic marker for NSCLC patients.

## Supporting Information

Table S1 **Clinical Characteristics of the NSCLC patients in the tissue dataset.** **Note:** “AD”, “SCC”, and “LCC” represent adenocarcinoma, squamous cell carcinoma, and large cell carcinoma, respectively.
(PDF)

Table S2 ***MHC*** **genes list detected in the NSCLC tissue dataset.** **Note:** The *MHC* genes family, which are also called human leukocyte antigen (*HLA*), is mainly divided into two subgroups: class I, class II; *MHC* I genes present antigens to the TCRs of CTL cells and *MHC* II genes present antigens to the TCRs of Th cells. The genes were clustered using hierarchical clustering by Euclidean distance and the order was consistent with that displayed in the Fig. 3A.
(PDF)
