# Supplementary material for: Deconvolution of the Gene Expression Profiles of Valuable Banked Blood Specimens for Studying the Prognostic Values of Altered Peripheral Immune Cell Proportions in Cancer Patients
Source: PLoS One. 2014 Jun 24;9(6):e100934. doi: 10.1371/journal.pone.0100934 (PMC4069164; doi:10.1371/journal.pone.0100934)
Supplement: Table S1 — Clinical Characteristics of the NSCLC patients in the tissue dataset. Note: “AD”, “SCC”, and “LCC” represent adenocarcinoma, squamous cell carcinoma, and large cell carcinoma, respectively. (PDF) [file pone.0100934.s001.pdf]

Table S1. Clinical Characteristics of the NSCLC patients in the tissue dataset

**Variable**	**Patients [N]**	**[%]**
**All**	139	100
**Histological subtype**		
AD	82	59
SCC	34	25
LCC	18	13
NSCLC	5	3
**Gender**		
Male	97	70
Female	42	30
**Stage**		
I	72	52
II & III	67	48
**Age (years)**		
<63	66	47
≥63	73	53
**Chemotherapy**		
No	139	100

Note: "AD", "SCC", and "LCC" represent adenocarcinoma, squamous cell carcinoma, and large cell carcinoma, respectively.
