# Supplementary material for: Deconvolution of the Gene Expression Profiles of Valuable Banked Blood Specimens for Studying the Prognostic Values of Altered Peripheral Immune Cell Proportions in Cancer Patients
Source: PLoS One. 2014 Jun 24;9(6):e100934. doi: 10.1371/journal.pone.0100934 (PMC4069164; doi:10.1371/journal.pone.0100934)
Supplement: Table S2 — MHC genes list detected in the NSCLC tissue dataset. Note: The MHC genes family, which are also called human leukocyte antigen (HLA), is mainly divided into two subgroups: class I, class II; MHC I genes present antigens to the TCRs of CTL cells and MHC II genes present antigens to the TCRs of Th cells. The genes were clustered using hierarchical clustering by Euclidean distance and the order was consistent with that displayed in the Fig. 3A. (PDF) [file pone.0100934.s002.pdf]

Table S2. MHC genes list detected in the NSCLC tissue dataset

**No.**	**Symbol**	***MHC* class**
**1**	*HLA-DOB*	II
**2**	*HLA-E*	I
**3**	*HLA-C*	I
**4**	*HLA-B*	I
**5**	*HLA-G*	I
**6**	*HLA-A*	I
**7**	*HLA-F*	I
**8**	*HLA-DQA2*	II
**9**	*HLA-DQA1*	II
**10**	*HLA-DRA*	II
**11**	*HLA-DRB5*	II
**12**	*HLA-DQB1*	II
**13**	*HLA-DRB1*	II
**14**	*HLA-DMA*	II
**15**	*HLA-DPB1*	II
**16**	*HLA-DPA1*	II
**17**	*HLA-DMB*	II
**18**	*HLA-DOA*	II

Note: The *MHC* genes family, which are also called human leukocyte antigen (*HLA*), is mainly divided into two subgroups: class I, class II; *MHC* I genes present antigens to the TCRs of CTL cells and *MHC* II genes present antigens to the TCRs of Th cells. The genes were clustered using hierarchical clustering by Euclidean distance and the order was consistent with that displayed in the Fig. 3A.
